# Glycocalyx components affect platelet function, whole blood coagulation, and fibrinolysis: an in vitro study suggesting a link to trauma-induced coagulopathy

**DOI:** 10.1186/s12871-021-01300-1

**Published:** 2021-03-19

**Authors:** Martin W. Britten, Laura Lümers, Kenji Tominaga, Jürgen Peters, Daniel Dirkmann

**Affiliations:** 1 Klinik für Anästhesiologie & Intensivmedizin, University of Duisburg-Essen & University Hospital of Essen, Hufelandstr. 55, 45122 Essen, Germany; 2 Present Address: Department of Anesthesiology and Critical Care Medicine, Fukuoka University School of Medicine, Fukuoka, Japan

**Keywords:** Glycosaminoglycans, Shock, Syndecan-1, Thrombomodulin, Versicans

## Abstract

**Background:**

The mechanisms of trauma induced coagulopathy (TIC) are considered multifactorial. Amongst others, however, shedding of the endothelial glycocalyx resulting in increased concentrations of glycocalyx fragments in plasma might also play a role. Thus, we hypothesized that shedded glycocalyx components affect coagulation and may act as humoral mediators of TIC.

**Methods:**

To investigate effects of heparan sulfate, chondroitin sulfate, syndecan-1, versican, and thrombomodulin we added these fragments to in vitro assays of whole blood from healthy volunteers to yield concentrations observed in trauma patients. Platelet function, whole blood coagulation, and fibrinolysis were measured by standard coagulation tests, impedance aggregometry (IA), and viscoelastic tests (VET). To assess dose-response relationships, we performed IA with increasing concentrations of versican and VET with increasing concentrations of thrombomodulin.

**Results:**

Intrinsically activated clotting times (i.e., activated partial thromboplastin time and intrinsically activated VET with and without heparinase) were unaffected by any glycocalyx fragment. Thrombomodulin, however, significantly and dose-dependently diminished fibrinolysis as assessed by VET with exogenously added rt-PA, and increased rt-PA-induced lysis Indices after 30 (up to 108% of control, *p* <  0,0001), 45 (up to 368% of control, *p* <  0,0001), and 60 min (up to 950% of control, *p* <  0,0001) in VET. Versican impaired platelet aggregation in response to arachidonic acid (up to − 37,6%, *p* <  0,0001), ADP (up to − 14,5%, *p* <  0,0001), and collagen (up to − 31,8%, *p* <  0,0001) in a dose-dependent manner, but did not affect TRAP-6 induced platelet aggregation. Clotting time in extrinsically activated VET was shortened by heparan sulfate (− 7,2%, *p* = 0,024), chondroitin sulfate (− 11,6%, *p* = 0,016), versican (− 13%, *p* = 0,012%), and when combined (− 7,2%, *p* = 0,007).

**Conclusions:**

Glycocalyx components exert distinct inhibitory effects on platelet function, coagulation, and fibrinolysis. These data do not support a ‘heparin-like auto-anticoagulation’ by shed glycosaminoglycans but suggest a possible role of versican in trauma-induced thrombocytopathy and of thrombomodulin in trauma-associated impairment of endogenous fibrinolysis.

## Background

Trauma and hemorrhagic shock are among the leading causes of death and morbidity worldwide [1]. In up to one third of trauma patients, hemorrhagic shock is complicated by trauma-induced coagulopathy (TIC), an independent risk factor for mortality [2]. The etiology of TIC is considered multifactorial [3] and alterations in platelet function [4], coagulation [5], and fibrinolysis [6] have been suggested to play major roles. Fibrinolysis, in particular, may be massively increased, leading to hyperfibrinolytic bleeding, or is endogenously suppressed, leading to ‘fibrinolysis shutdown’. Both conditions are associated with an increased mortality [7]. At least in part, some of these alterations can be explained or are aggravated by acidosis, hypothermia, hemodilution, consumption and loss of procoagulant factors and of platelets [3]. However, the mediators leading to acquired platelet dysfunction, alterations in thrombin generation, and the frequently observed pro- or antifibrinolytic states (hyperfibrinolysis vs. lysis shutdown) are largely unknown.

The endothelial glycocalyx layer contains various proteoglycans and glycoproteins and some of those, by binding anticoagulant mediators such as antithrombin III, heparin cofactor II, thrombomodulin, or tissue factor pathway inhibitor (TFPI) exert anticoagulant properties [8]. Injury to the glycocalyx, as demonstrated by increased circulating concentrations of its fragments, has been observed in trauma patients and this is associated to mortality [9, 10]. Proteoglycans consist of a protein core with covalently linked glycosaminoglycans (GAG). Even though the glycocalyx composition differs according to the type of tissue, the main GAG are heparan sulfates, chondroitin sulfates, and hyaluronan, while syndecan-1 is among the most common proteoglycans. The latter is also one of the best-established markers of glycocalyx shedding and attains plasma concentrations of up to 477 ng/ml in trauma patients compared to median 31.6 ng/ml in healthy controls [10]. Heparan sulfate is increased up to 185 ng/ml in trauma patients compared to 133.9 ng/ml in healthy controls [10]. As hyaluronan has not been shown to be increased in trauma [10], we did not include it in our experiments. Chondroitin sulfate has a physiological concentration of 5.17 μg/ml [11], but its concentration almost doubles in trauma patients [10]. Thrombomodulin is an endothelial protein and thrombin receptor which regulates hemostasis. Thus, its dissociation from the endothelium may lead to a systemic effect on coagulation and increase in its systemic concentrations is an independent predictor of trauma associated kidney failure and mortality [12, 13]. Soluble thrombomodulin shows concentrations of up to 9.4 ng/ml in trauma patients [9, 12]. Versican is a large proteoglycan and known as a critical mediator in inflammation, vascular injury, and wound healing [14]. Although it has not yet been related to trauma, its known “versatile” structure and functions made it logical to examine its role. While some glycosaminoglycans are only found in specific tissues, versican is found especially in vessel walls in a broad variety of tissues [15]. Therefore, even though we were unable to identify specific versican concentrations in trauma patients, it is likely to be shedded under those conditions. Accordingly, we included versican in our experiments assuming a 10-fold increase in plasma concentrations, in analogy to syndecan-1.

Therefore, we hypothesized that glycocalyx components such as syndecan-1, heparan sulfate, chondroitin sulfate, thrombomodulin, and versican may act as mediators of TIC and hence examined in vitro their individual and combined in vitro effects on platelet function, whole blood coagulation, and fibrinolysis.

## Methods

Following approval of the local ethics committee (Medical faculty of the University of Duisburg-Essen, Ethics Committee, no. 17–7772-BO) and written informed consent, blood (total 39,8 ml, drawn via 21 g cannula, and with the first sample tube discarded to avoid contamination with thromboplastin) from 15 healthy volunteers (9 male, 6 female), median age 34 years (range: 27 to 41) was withdrawn into hirudin anticoagulated and citrate anticoagulated test tubes (Sarstedt, Nürnbrecht, Germany, product code 04.1959.001 for impedance aggregometry, 04.1919 for VET). All volunteers had an unremarkable bleeding history and denied the intake of any medication during the past weeks.

Glycocalyx fragments dissolved in Phosphate Buffered Saline (PBS) were added to the fresh citrated blood samples so as to achieve trauma-equivalent concentrations [10]. Specifically, final concentrations were 180 ng/ml for heparan sulfate (product no. H4777, Sigma-Aldrich, St. Louis, USA), 12 μg/ml for chondroitin sulfate (product no. C 9819, Sigma-Aldrich, St. Louis, USA), 200 ng/ml for syndecan-1 (catalogue no. 2780-SD, R&D Systems, Minneapolis, USA), and 7 ng/ml for thrombomodulin (product no. ab98989, Abcam, Cambridge, UK), respectively. Since we could not identify from the literature any versican concentration typical for trauma patients, we assumed (analogous to syndecan-1) a 10-fold increase of its plasma concentration. Since plasma versican concentrations in healthy subjects are 95 ng/ml on average, versican (product code 230–00833, RayBiotech, Norcross, USA) was added to achieve a final concentration of 950 ng/ml [16]. An additional sample was prepared using the concomitant application of all substances in their respective final concentrations outlined above. To serve as a control, another sample was prepared with phosphate buffered saline (PBS) only. To avoid effects of different dilutions, the volume added to all tubes was standardized to 10% of the total volume in all experimental series and assays including the control samples.

Since we identified significant effects of versican and thrombomodulin on fibrinolysis resp. platelet aggregation in our first series of experiments, we also assessed possible dose-dependency of these effects. Therefore, we performed a second series of experiments to yield various final concentrations of versican (vehicle, 475 ng/ml, 950 ng/ml, and 1900 ng/ml) and of thrombomodulin (vehicle, 3,5 ng/ml, 7 ng/ml, and 14 ng/ml). We chose these concentrations below and above those typical for trauma patients to not only assess any dose-dependency, but also to examine effects of minor, even more commonly found concentrations. Again, blood was withdrawn and prepared from 22 healthy volunteers (11 male, 11 females, age: median 35 years, range 21–59), as described above.

### Measurements

To assess the impact of the interventions on coagulation, standard coagulation tests, viscoelastic tests (VET), and impedance aggregometry (IA) were performed on each sample.

Prothrombin time (PT, as International Standardized Ratio, INR) and activated partial thromboplastin time (aPTT) were performed according to standard procedures in the hospital’s central laboratory.

VET was performed by rotational thromboelastometry and using in parallel four ROTEM® delta devices (Werfen GmbH, Munich, Germany) using five different assays and activators, i.e., extrinsic activation (EXTEM®) with tissue factor (TF), intrinsic activation (INTEM®) with ellagic acid, extrinsic activation with TF and abolition of platelet function using cytochalasin D so as to assess fibrin polymerization (FIBTEM®), and intrinsic activation after inactivation of any heparin and heparinoids by heparinase (HEPTEM®). The latter four assays were performed according to the specifications of the manufacturer, while a second INTEM® assay was modified to examine interactions of the glycocalyx fragments with fibrinolysis. Standardized tests to assess in vitro antifibrinolytic effects have not yet been established. However, previous work used extrinsically activated rotational thromboelastometry (EXTEM) with added rt-PA to address such questions [17–22]. As we expected heparinoid effects evoked by some of the tested glycocalyx components and the EXTEM-reagent contains polybrene (hexadimethrine bromide), an inhibitor of heparin, we modified this approach with an intrinsically activated test (INTEM) and using the same final rt-PA (Actilyse®, Boehringer Ingelheim Pharma, Ingelheim am Rhein, Germany) concentration of 100 ng/ml. Even though most measurements were taken almost simultaneously on up to 4 ROTEM analyzers, some samples of our first series of experiments were measured with a time delay of approximately up to 140 min. However, time effects on citrated blood samples for viscoelastic measurements are unlikely: stability of samples has been demonstrated for up to 6 h when using rotational thromboelastometry [23]. Additionally, to avoid any bias by the timing of experiments, we performed viscoelastic tests of our first series of experiments in a random order. The following ROTEM® variables were assessed: clotting time (CT), clot formation time, maximum clot firmness (MCF), and clot lysis index 45 min (LI45) after initial clotting. All assays were run for at least 60 min after initiation of clotting (CT).

Impedance aggregometry (IA) was performed with an analogous protocol on hirudin anticoagulated blood samples. Hirudin was chosen as the anticoagulant as recommended by the manufacturer. After preparation of the assays with the glycocalyx fragments or PBS as a control, IA was performed on two impedance aggregometry devices (Multiplate® Analyzer, Roche, Germany). Platelets were activated with arachidonic acid (ASPItest®), adenosine-diphosphate (ADPtest®), or thrombin receptor activator peptide-6 (TRAP-6) (TRAPtest®), according to the manufacturer’s instructions. Results were assessed as area under the curve (AUC).

For the second series of experiments, IA after activation with arachidonic acid, ADP, TRAP-6, and collagen (COLtest®) was performed with versican in final concentrations of 475 ng/ml, 950 ng/ml, 1900 ng/ml, and vehicle. Again, VET after intrinsic activation and with addition of 100 ng/ml rt-PA to the test cups was performed with thrombomodulin in final concentrations of 3,5 ng/ml, 7 ng/ml, 14 ng/ml, and vehicle. We specifically examined clot lysis indices, representing the remaining clot strength after 30, 45 and 60 min (LI30, LI45, LI60) to characterize the antifibrinolytic effect.

#### Statistics

Data were analyzed using Prism 6 (Version 6.0b for Mac OS X, GraphPad Inc., San Diego, USA). Normal distribution of data could not be demonstrated using a Kolmogorov–Smirnov test with the Dallal and Wilkinson approximation to Lilliefor’s method. Accordingly, non-parametric Friedman tests with Dunn’s adjustment of the α-error for multiple testing were used. Results are shown as median (25th/75th percentile) and mean percentage deviations compared to the respective controls.

## Results

### Standard laboratory tests

We could not demonstrate a significant effect of any of the glycocalyx components on aPTT nor on prothrombin time (INR) **(**Table 1**)**.

**Table 1:** Results using standard coagulation tests, viscoelastic testing (VET; ROTEM®), and impedance aggregometry (IA; Multiplate®). Data are presented as median (25./75. percentile). *P*-values refer to comparisons with control. P-values of HEPTEM CT in comparison to corresponding INTEM CT. *P* < 0.05 marked with asterisk*. Non-significant P-values > 0,05 are referred to as ‘n. s’

	Control	Syndecan-1	Thrombo-modulin	Heparan Sulfate	Chondroitin Sulfate	Versican	Combination
**aPTT (s)**	30,1 (28,5/33,7)	28,8 (28,4/31,6)	30,2 (28,8/34)	28,9 (28/31,9)	29,1 (28,4/33,9)	29,8 (28,9/32,3)	32,2 (29,6/33)
*P value*		*n. s.*	*n. s.*	*n. s.*	*n. s.*	*n. s.*	*n. s.*
**INR**	1065 (1033/1,11)	1055 (1,02/1193)	1,06 (1005/1,09)	1,02 (1,01/1103)	1085 (1,04/1148)	1,09 (1,02/1208)	1,1 (1008/1158)
*P value*		*n. s.*	*n. s.*	*n. s.*	*n. s.*	*n. s.*	*n. s.*
**EXTEM CT (s)**	69 (64/76)	67 (62/73)	68 (62/71)	64 (58/69)*	61 (58/68)*	60 (57/67)*	64 (57/68)*
*P value*		*n. s.*	*n. s.*	*0.0244*	*0.0162*	*0.0122*	*0.0068*
**EXTEM MCF (mm)**	62 (58/65)	61 (58/66)	61 (58/64)	60 (58/66)	61 (59/64)	62 (58/65)	61 (59/64)
*P value*		*n. s.*	*n. s.*	*n. s.*	*n. s.*	*n. s.*	*n. s.*
**EXTEM LI45 (%)**	96 (92/98)	97 (92/98)	96 (91/97)	97 (92/97)	97 (94/98)	96 (95/98)*	97 (94/98)*
*P value*		*n. s.*	*n. s.*	*n. s.*	*n. s.*	*0.001*	*0.0018*
**INTEM CT (s)**	211 (194/257)	204 (180/229)	185 (173/213)	193 (172/206)	193 (165/233)	203 (166/238)	188 (180/208)
*P value*		*n. s.*	*n. s.*	*n. s.*	*n. s.*	*n. s.*	*n. s.*
**INTEM MCF (mm)**	60 (57/63)	58 (56/61)	58 (57/61)	57 (56/63)	60 (56/61)	60 (57/62)	61 (57/62)
*P value*		*n. s.*	*n. s.*	*n. s.*	*n. s.*	*n. s.*	*n. s.*
**INTEM LI45 (%)**	94 (91/97)	94 (91/96)	93 (90/96)	94 (90/96)	94 (91/96)	95 (92/97)	95 (91/97)
*P value*		*n. s.*	*n. s.*	*n. s.*	*n. s.*	*n. s.*	*n. s.*
**HEPTEM CT (s)**	194 (172/219)	197 (166/208)	199 (190/210)	197 (164/227)	192 (174/210)	192 (175/213)	201 (181/224)
*P value (*vs. *INTEM CT)*		*n. s.*	*n. s.*	*n. s.*	*n. s.*	*n. s.*	*n. s.*
**FIBTEM CT (s)**	68 (62/75)	64 (59/73)	64 (61/70)	64 (59/66)	59 (56/60)	60 (55/66)	61 (58/67)
*P value*		*n. s.*	*n. s.*	*n. s.*	*n. s.*	*n. s.*	*n. s.*
**FIBTEM MCF (mm)**	13 (11/17)	13 (10/16)	14 (10/16)	12 (11/15)	13 (11/16)	13 (11/17)	13 (11/17)
*P value*		*n. s.*	*n. s.*	*n. s.*	*n. s.*	*n. s.*	*n. s.*
**FIBTEM LI45 (%)**	100 (99/100)	100 (100/100)	100 (100/100)	100 (100/100)	100 (99/100)	100 (100/100)	100 (100/100)
*P value*		*n. s.*	*n. s.*	*n. s.*	*n. s.*	*n. s.*	*n. s.*
**rt-PA INTEM LI30 (%)**	77 (44/86)	77 (56/92)	94 (85/97)*	76 (67/91)	80 (73/93)	80 (71/88)	95 (89/97)*
*P value*		*n. s.*	*n. s.*	*n. s.*	*n. s.*	*n. s.*	*< 0.0001*
**rt-PA INTEM LI45 (%)**	2 (1/12)	4 (1/12)	41 (19/51)*	8 (4/30)	9 (5/28)	9 (4/22)	35 (23/64)*
*P value*		*n. s.*	*< 0.0001*	*n. s.*	*n. s.*	*n. s.*	*< 0.0001*
ADPtest (AU*min)	900 (815/983)	852 (759/943)	781 (607/946)	882 (613/925)	844 (735/939)	739 (648/832)*	734 (510/784)*
*P value*		*n. s.*	*n. s.*	*n. s.*	*n. s.*	*0.0233*	*0.0005*
ASPItest (AU*min)	935 (835/1111)	956 (901/1024)	939 (815/1093)	931 (863/1032)	1010 (917/1101)	757 (688/876)*	767 (589/897)*
*P value*		*n.s.*	*n.s.*	*n.s.*	*n.s.*	*0.0458*	*0.0014*
TRAPtest (AU*min)	1027 (1187/1257)	1060 (1090/1199)	1022 (1125/1279)	938 (1155/1279)	993 (1142/1217)	995 (1153/1268)	974 (1063/1196)
*P value*		*n. s.*	*n. s.*	*n. s.*	*n. s.*	*n. s.*	*n. s.*

### Viscoelastic testing (VET)

CT after intrinsic activation (INTEM®) was unaltered in all assays and addition of heparinase (HEPTEM®) did not shorten CTs (Table 1). CT after extrinsic activation (EXTEM®) was significantly shorter with heparan sulfate (− 7,2%, *p* = 0.0244), chondroitin sulfate (− 11,6%, *p* = 0.0162), versican (− 13%, *P* = 0,0122), and with their combined application (− 7,2%, *p* = 0.0068) (Table 1). Fibrin polymerization was unaltered, since FIBTEM® assays did not show significant differences in clot firmness, clotting time, or lysis indices (Table 1).

Fibrinolysis, as quantitatively expressed by the LI45%, was markedly attenuated by thrombomodulin and by the combined application of all glycocalyx components when tested in intrinsically activated rotational thromboelastometry (INTEM®) with rt-PA addition (Table 1). A concentration of 7 ng/ml thrombomodulin compared to controls (LI45 2% (1/12)), increased LI45 by as much as 41% (19/51) and the combined application of all glycocalyx fragments with thrombomodulin increased LI45 to 35% (23/64). The other test substances had no significant effect on LI45 **(**Table 1**)**.

**Fig. 1:**
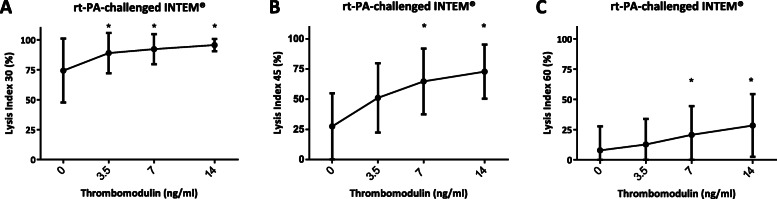
Effects of increasing thrombomodulin-concentrations on fibrinolysis. VET with intrinsic activation and rt-PA-challenge (100 ng/ml) revealed increasing Lysis Indices after (**a**) 30, (**b**) 45, and (**c**) 60 min, representing a dose-dependent antifibrinolytic effect. Means ±SD, *P* < 0.05 marked with asterisk*

This antifibrinolytic effect was confirmed to be dose-dependent (Fig. 1). Thrombomodulin in a concentration of 3,5 ng/ml increased LI30 (95% compared to control 84%, *P* = 0,0307), but not LI45 or LI60. In concentrations exceeding 7 ng/ml thrombomodulin also increased LI45 (73%, compared to control 62%, *p* < 0,0001), and LI60 (12% compared to control 2%, *p* = 0,0004).

### Impedance aggregometry (IA)

Significant inhibition of platelet aggregation by versican and the combined application of all glycocalyx fragments was observed in response to platelet activation by ADP and arachidonic acid. Specifically, versican (950 ng/ml) decreased platelet aggregation (AUC*min) by 17,9% (*p* = 0.0233) in the ADPtest and by 19% (*p* = 0,0458) in the ASPItest. The combination of all glycocalyx components decreased aggregation by 18,4% (*p* = 0.0005) in the ADPtest and by 18% (*p* = 0.0014) in the ASPItest, presumably due to the versican component. Platelet aggregation was unchanged by the other glycocalyx components and platelet function after TRAP-6 activation was unaltered in all tests (Table 1).

**Fig. 2:**
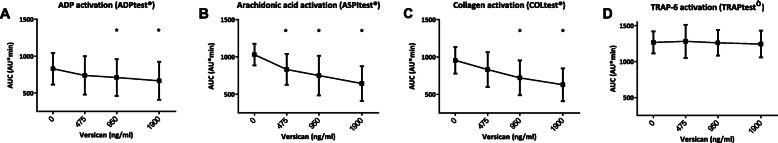
Effects of increasing versican-concentrations on platelet aggregation, as measured by impedance aggregometry (IA) with platelet activation by (**a**) ADP, (**b**) arachidonic acid, (**c**) collagen, and (**d**) TRAP-6. Versican dose-dependently inhibits platelet aggregation in response to ADP, arachidonic acid, and collagen, but not TRAP-6. Means ± SD, P < 0.05 marked with asterisk*

The second series of experiments confirmed a dose-dependent inhibition of platelet aggregation after activation with ADP (− 12,5%, p = 0,0033) and collagen (− 23,4%, *p* < 0,0001) by versican concentrations exceeding 950 ng/ml and after activation with arachidonic acid (− 19,7%, p = 0,0218) by versican concentrations exceeding 475 ng/ml (Fig. 2). In line with the first experimental series, platelet function after activation with TRAP-6 remained unaltered.

## Discussion

The data from this in vitro study suggest a possible role for endothelial glycocalyx components as mediators in TIC since different glycocalyx fragments showed distinct dose-dependent effects on fibrinolysis, platelet function, and tissue factor-induced clotting time. Thrombomodulin exerted a potent antifibrinolytic effect, whereas versican significantly impaired platelet aggregation. Furthermore, several glycocalyx molecules (i.e., heparan sulfate, chondroitin sulfate, versican) shortened clotting time in extrinsically activated assays. In contrast, our results do not support relevant heparin-like effects of the examined glycocalyx fragments, at least not in concentrations commonly found in trauma patients [10].

The understanding of TIC has advanced from the so-called “lethal triad of trauma”, i.e., hypothermia, acidosis, and coagulopathy, to an acute derangement of hemostatic processes. Amongst others, alterations in thrombin generation, mechanical clot firmness, platelet dysfunction, and fibrinolysis have all been described [3, 24]. However, the mechanisms contributing to these alterations have not been elucidated.

Disruption of the endothelial glycocalyx both by direct and indirect damage has been reported in trauma patients and traumatic “endotheliopathy” predicts mortality [12]. Furthermore, since the endothelial glycocalyx physiologically regulates coagulation, glycocalyx fragments are likely to be mediators of a coagulopathy when released into the blood stream. Syndecan-1 is among the best-established markers of glycocalyx shedding, the plasma concentrations of the latter, as well as of heparan sulfate, chondroitin sulfate and soluble thrombomodulin are considerably elevated in trauma patients [9, 10].

Alterations of the fibrinolytic system also have an impact on mortality in trauma patients. Both, a pathologically increased (hyperfibrinolysis) as well as a downregulated fibrinolysis (“fibrinolytic shutdown”) correlate positively with mortality in trauma patients [6, 7]. Hyperfibrinolysis in trauma patients mainly results from shock-induced release of t-PA from endothelial cells of hypoperfused tissues, but may be aggravated by decreased concentrations of plasminogen activator inhibitor 1 (PAI-1) [20]. In contrast, the mechanisms of a downregulated endogenous fibrinolysis, commonly termed ‘fibrinolytic shutdown’, are far less understood. Plasmatic alpha-2-macroglobuline, C1-Inhibitor, and alpha-2-antiplasmin as well as platelet-derived PAI-1 release are considered potential factors [25, 26]. Our results suggest an additional role of soluble thrombomodulin (sTM) as an inhibitor of fibrinolysis in TIC, since the concentration of exogenously added sTM used in our assays was similar to that in trauma patients [12]. In the latter study, increased sTM was associated with an increased 7-day and 28-day mortality. As our results even show an effect for low concentrations of sTM, a major part of trauma patients may be affected by sTM-induced inhibition of fibrinolysis. Moreover, as we observed a dose-dependent effect, the sTM-concentration and its proportion to concentrations of other influencing factors balancing the fibrinolytic system may contribute to the determination whether the patient will exhibit a suppressed fibrinolysis (“fibrinolytic shutdown”) or a hyperfibrinolytic state after trauma.

Diminished platelet function is a risk factor for massive transfusion and mortality in severely injured patients [4]. It affects about 46% of trauma patients leading to a state of acquired platelet dysfunction, that commonly persists for as long as 96 h [27]. Furthermore, decreased platelet function, as assessed by thromboelastographic platelet mapping assays, is associated with increased fibrinolysis [25]. Recently, trauma-induced thrombocytopathy was shown to be induced by yet unknown plasmatic mediators [28].

In our study, soluble versican significantly inhibited platelet aggregation in response to ADP, arachidonic acid, and collagen in a dose-dependent manner. Thus, versican may be one of the unknown plasmatic mediators of trauma-induced thrombocytopathy. Versican is a large proteoglycan that has been described to promote platelet adhesion and to induce platelet aggregation when associated with collagen [29, 30]. In this respect, our results, rather indicating an anti-platelet effect of versican, seem to be somewhat contradictory. However, we speculate that circulating versican lacks its anchoring collagen structure but still interacts with platelets, thereby hindering platelet aggregation. Recently, the presence of shock rather than the degree of injury has been suggested as the common denominator resulting in the release of mediators for platelet inhibition [31]. This finding further supports our hypothesis of glycocalyx components being partly responsible for trauma-induced platelet dysfunction since shock, not injury itself, evokes endothelial disruption and hence shedding of glycocalyx components [32]. As versican dose-dependently inhibited platelet aggregation, a role in trauma-induced platelet dysfunction seems suggestive.

Versican did not influence platelet activation via the TRAP-6-pathway. However, TRAP-6-mediated platelet activation via the thrombin-receptor-pathway is one of the most powerful stimuli to activate platelets and it is not uncommon for drugs with established antiplatelet activity to still allow in vitro activation by TRAP-6. Accordingly, attenuated platelet aggregation after stimulation with ADP, arachidonic acid, and collagen indicates a relevant inhibition of platelet function.

With respect to fibrinolysis, we did not find any effects of versican induced platelet inhibition on fibrinolysis, since thromboelastometry data with exogenous rt-PA were unaffected. This finding is interesting, as decreased resistance against hyperfibrinolysis and up to the extreme of hyperfibrinolysis is a key component of TIC [21, 26, 33]. Platelets confer resistance to fibrinolysis by providing antifibrinolytic molecules such as plasminogen-activator-inhibitor-1 (PAI-1) or α2-antiplasmin and by promoting clot firmness [21, 22, 34]. As we found evidence for versican-induced platelet dysfunction, one could therefore suspect versican to induce decreased fibrinolytic resistance. However, our experiments did not confirm such a finding, possibly due to the small extent of platelet inhibition.

Versican, chondroitin sulfate, and heparan sulfate significantly shortened clotting times in extrinsically (i.e., tissue factor) activated viscoelastic assays, likely reflecting accelerated thrombin generation. This effect of versican may be explained by inhibitory effects of the versican core protein on TFPI-1 [29]. Moreover, we also observed shortening of the extrinsic CT after incubation with heparan sulfate and chondroitin sulfate. One might thus speculate, whether the core protein of heparan sulfate shares similar properties regarding the suppression of TFPI-1. Nevertheless, our observation of shortened CTs are consistent with prior observations since an increased endogenous thrombin potential has been described in trauma patients, despite decreased plasma concentrations of prothrombin [35]. The latter authors concluded, that some kind of circulating procoagulant and decreased anticoagulant might be responsible. Our data indicate a potential role for circulating glycocalyx components as drivers for increased thrombin generation, possibly by inhibition of TFPI-1. As the concomitant application of glycocalyx fragments did not result in a further shortening of clotting time, there appears to be no synergistic effect of these fragments on thrombin generation. The reason for this is unknown, but it may support the speculative inhibition of TFPI-1 as this effect will reach its limit as soon as inhibition is complete. In case a single glycocalyx fragment in the given concentration already suppresses TFPI-1 completely, others will be unable to further increase thrombin generation.

In this context, it also seems noteworthy that endogenous heparin-like anticoagulant substances, as suggested by others [36], likely would have resulted in decreased thrombin generation. Our data question relevant heparin-like anticoagulatory effects of the glycocalyx fragments examined along several arguments: (1) CTs of heparin sensitive, intrinsically activated assays (i.e., aPTT and INTEM®) were unaffected by all fragments. (2) Addition of heparinase to another intrinsically activated assay (HEPTEM®) would have abolished any prolongation of clotting time induced by such “heparinoids”, but clotting times in paired assays with and without heparinase did not differ significantly (Table 1). Prior in vitro studies have demonstrated prolongation of intrinsically activated CTs after incubation with heparan sulfate, dermatan sulfate, and chondroitin sulfate, and abolition of this effect by heparinase for the latter two fragments [36]. However, we used clinically relevant concentrations as reported in severely injured patients [10], while the latter authors examined concentrations of a 50-fold greater magnitude. Even the concomitant application of glycocalyx fragments was unable to evoke a heparin-like anticoagulatory effect, most likely because of the major difference between these concentrations. Thus, even though there may be an in vitro ‘heparinoid’ effect of supraphysiologic concentrations of glycocalyx fragments, our results question its clinical relevance without other influencing factors.

Our study has obvious limitations. As the expected effects of glycocalyx components were unknown in modality and extent, we were unable to calculate an a priori power analysis before our experiments. Even though the small subject number has been sufficient in previous studies [18, 19, 37], we cannot fully exclude additional minor effects, that may not have been detected under the circumstances outlined. Most importantly, by addition of glycocalyx molecules to the assays we altered only one condition in our experiments. This neglects other trauma-associated alterations, possibly rendering an already impaired coagulopathic system even more vulnerable to anticoagulatory agents. Acidosis, hemodilution, and hypothermia are only some examples of such a noxious environment, in which glycocalyx fragments might have different and possibly even greater effects. However, we deliberately chose to perform our experiments in normothermic, only 10% diluted samples and at a physiologic pH so as to distinguish other influences on coagulation from the effects of glycocalyx fragments. Moreover, it is uncertain, whether an in vitro study fully reflects an in vivo situation. Although we performed the VET and IA in whole blood samples, this means leaving out a potential interplay with other organs or endothelial cells. It must remain speculative, whether the concentrations of circulating glycocalyx components and coagulation factors used in this study and measured in the blood stream by others [9, 10, 12, 38, 39] strictly reflect the respective conditions in localized compartments during TIC. However, even though local environments may be crucial for coagulation pathophysiology, it is almost impossible to simulate in vitro those environments with all its interactions. In fact, our in vitro findings of glycocalyx components may even underestimate the effects in a localized compartment.

Furthermore, as there is no validated in vitro whole blood test for an antifibrinolytic effect, we used a rt-PA-challenged INTEM-assay as a modification of a previously published method [17–22]. For interpretation of test results, we minimized bias by any assay-inherent variations using randomized measurement procedures, a low coefficient of variation, and a second series of experiments demonstrating a clear dose-response relationship. Even though the still non-standardized nature of the rt-PA-challenged INTEM-assay may represent a study limitation, we, therefore, consider this unlikely.

The pathophysiology of trauma-induced platelet impairment is incompletely understood and, in general, platelet activation must be distinguished from platelet aggregation. Recently, trauma-induced platelet dysfunction has been characterized as a malfunction of adhesion, aggregation, and contraction despite preserved flow-cytometric markers of platelet activation [40]. Therefore, we did not examine by flow cytometry platelet surface markers of activation. Impedance aggregometry in whole blood can be influenced by very low platelet counts in the sample [41]. Even though we used only 10% diluted blood of healthy volunteers which usually have platelet counts *above* 200/nl [42], we cannot fully exclude a thrombopenic sample. However, asymptomatic thrombocytopenia in our healthy probands is very unlikely and we also can rule out severe thrombocytopenia since maximum clot firmness (MCF) in extrinsically activated rotational thromboelastometry (EXTEM and FIBTEM) was not decreased. Second, as interventions were always compared to a control sample from the same volunteer, we doubt that even a thrombopenic volunteer would have changed results. Despite these theoretical limitations, our findings provide new data on the action of glycocalyx fragments on several features of hemostasis.

## Conclusions

Clinically relevant concentrations of various endothelial glycocalyx components significantly affect whole blood clotting time, platelet aggregation, and fibrinolysis, suggesting a role for these molecules in the development of TIC. The most noteworthy effect was the dose-dependent inhibition of platelet function by versican and a lysis shutdown evoked by soluble thrombomodulin in concentrations commonly found in trauma patients. Accordingly, our results provide new insights into possible mechanisms of TIC.

## Data Availability

The datasets used and/or analyzed during the current study are available from the corresponding author on reasonable request.

## Abbreviations

ADP: Adenosine diphosphate
aPTT: activated partial thromboplastin time
AUC: Area under the curve
CT: Clotting time
IA: Impedance aggregometry
INR: International Normalized Ratio
LI: Lysis index
PAI-1: Plasminogen activator inhibitor 1
PBS: Phosphate buffered saline
PT: Prothrombin time
ROTEM: Rotational thromboelastometry
rt-PA: recombinant tissue-type plasminogen activator
sTM: soluble Thrombomodulin
TF: Tissue factor
TFPI: Tissue factor pathway inhibitor
TIC: Trauma-induced coagulopathy
TRAP-6: thrombin receptor-activating peptide 6
VET: Viscoelastic tests
